# Mental health in primary care: Integration through in-service training in a South African rural clinic

**DOI:** 10.4102/phcfm.v10i1.1660

**Published:** 2018-05-24

**Authors:** Lucy Maconick, Louis S. Jenkins, Henriette Fisher, Anthony Petrie, Lynnie Boon, Hermann Reuter

**Affiliations:** 1 Improving Global Health, NHS Thames Valley and Wessex Leadership Academy, United Kingdom; 2 Department of Family and Emergency Medicine, Faculty of Health Sciences, University of Stellenbosch, South Africa; 3 Department of Family and Emergency Medicine, George Provincial Hospital, Eden and Central-Karoo districts, Western Cape Department of Health, South Africa; 4 Conville Community Health Clinic, George, Eden district, Western Cape Department of Health, South Africa; 5 Department of Psychiatry, George Provincial Hospital, Eden and Central-Karoo districts, Western Cape Department of Health, South Africa; 6 George sub-district, Division of Primary Health Care, University of Cape Town, South Africa

## Abstract

**Background:**

Integrating mental health into primary care is a global priority. It is proposed to ‘task-share’ the screening, diagnosis and treatment of common mental disorders from specialists to primary care workers. Key to facilitating this is training primary care workers to deliver mental health care. Mental health training in Africa shows a predominance of short-term, externally driven training programmes. Locally, a more sustainable delivery system was needed.

**Aim:**

The aim of the study was to develop and evaluate a locally delivered, long-term, in-service training programme to facilitate mental health care in primary care.

**Methods:**

This was a quasi-experimental study using mixed methods. The in-service training programme was delivered in weekly 1-h sessions by local psychiatry staff to 20 primary care nurses at the clinic over 5 months. The training was evaluated using quantitative data from participant questionnaires and analysis of the referrals from primary to specialist care. Qualitative data were collected via semi-structured interviews and 14 observed training sessions.

**Results:**

The training was feasible and well received. Referrals to the mental health nurse increased in quality and participants’ self-rated competence improved. Additional benefits included the development of supervision skills of mental health nurses and providing a forum for staff to discuss service improvement. The programme acted as a vehicle to pilot integration in one clinic and identify unanticipated barriers prior to rollout.

**Conclusions:**

Long-term, in-service training, using existing local staff had benefits to the integration of mental health into primary care. This approach could be relevant to similar contexts elsewhere.

## Background

Mental health has been recognised as a global priority. The World Health Assembly adopted the World Health Organization (WHO) Comprehensive Mental Health Plan 2013–2020 in 2013.^1,2^ One of the objectives of the plan stipulates that health systems should ‘provide comprehensive, integrated and responsive mental health and social care services in community-based settings’.^1^ With limited resources for mental health worldwide, a call has been made to task-share some traditional mental health services to other cadres of staff.^3,4,5^

In South Africa, there is increasing momentum to integrate mental health care into primary health care (PHC).^6^ One reason for this is the reality of too few mental health care practitioners, particularly in the public health sector (which serves 80% of the population) and especially in the rural areas.^7^ It is estimated that 75% of people with a mental health problem in South Africa do not receive treatment.^8^ Some unintended consequences of traditionally having a vertical mental health care service within PHC have also been observed, including a dichotomy of care and non-recognition of mental illness as a driver of many PHC complaints. This was suggested by a study of 18 856 consultations where headache, generalised body pains and other somatic complaints were frequent reasons for encounter in PHC clinics in South Africa, while no psychiatric illness appeared amongst the top 25 diagnoses.^9^ There is also a recognised impact of comorbid mental health on chronic health conditions. An analysis of the World Health Surveys reported that patients with one or more physical health complaints are significantly more likely to have depression than those without chronic physical disease. Those with depression comorbid with physical disease had the poorest health scores of all the disease combinations.^10^ There are benefits to physical health outcomes when depression is identified and treated. For example, treatment for depression has been strongly associated with improved outcomes from treatment for HIV with highly active antiretroviral therapy (HAART).^11^

Part of a proposed model of integration, in line with WHO, is to ‘task-share’ some mental health care with primary care workers in order to improve access to specialist psychiatry care within available resources.^12^ There are also some perceived secondary benefits, such as the reduction of stigma. The Practical Approach to Care Kit (PACK) or Primary Care 101 guidelines used in the Western Cape province provides a tool for primary care workers to perform screening, assessment and evidence-based treatment for patients with mental health complaints, before onward referral.^13^

A core ingredient for implementation of this model is the training of PHC workers to deliver quality mental health care. However, there is a paucity of literature on evaluating training strategies towards this end. A recent systematic review found a predominance of short, high-intensity training programmes, many delivered in partnership with expert tutors from high-income countries.^14^ A need for a pragmatic, feasible and sustainable model of integration and training for mental health care in PHC is needed for low-resource settings, such as the one described here.

We describe an in-service, long-term training programme delivered in a rural community health centre in South Africa by the local mental health team, aimed at improving task-sharing and integration of mental health into PHC. The programme aims to provide a sustainable model of training that can be rolled out without large numbers of additional human resources. The training sessions also aimed not only to improve knowledge of mental health, but also to provide a supervision system for PHC workers and a feedback mechanism for the mental health team to communicate with PHC staff.

Training in this model is provided predominantly by the mental health nurse working in the district. The programme includes provision of ‘training the trainers’ and educational mentoring for mental health nurses to develop skills in teaching and supervision. Fostering these skills in specialist teams will be important if ‘task sharing’ is to succeed. This aims to empower mental health nurses to improve their own service as well as being a more sustainable, long-term approach to short, externally driven courses.

## Methods

### Study design

Mixed methods were used in this quasi-experimental study to qualitatively and quantitatively evaluate the implementation of a mental health care training programme.

### Setting

The study was performed at Conville Community Health Centre in George, a rural town in the Eden district of South Africa. The centre is one of the 10 PHC facilities that serve a predominantly public sector population of about 200 000 people. The clinic is staffed by eight PHC nurses and three PHC doctors, a pharmacist, a dentist, a visiting physiotherapist and some sessional health staff. A mental health care nurse is allocated to the clinic from Monday to Friday (the clinic is closed after hours and weekends) and a consultant psychiatrist visits once a week. The clinic also has access to the one district psychologist. This clinic was chosen as the study site as it has a high number of mental health care consultations (200–300 per month) and these were performed almost exclusively by the mental health nurse. Once patients screen positive for a mental illness by the PHC staff, they are referred to the mental health care nurse. Assessment of patients prior to referral to the mental health nurse was perceived as very limited.

### Study population

All primary care workers at the clinic were invited to the training. In addition, some staff from neighbouring smaller clinics who referred patients to the clinic also attended the training. The participants included primary care trained nurses, health promoters and professional nurses. We took an inclusive approach with the aim of fostering collaborative care and encourage interprofessional communication in the clinic. Fourteen participants were included in the evaluation. Twenty participants attended at least one training session. Staff were excluded from the evaluation if they missed more than three training sessions.

### The intervention

The PACK guidelines were used as the core content of the training. This package of PHC symptoms was developed by the Knowledge Translation Unit of the University of Cape Town, and has extensively been tested and implemented in various settings in South Africa and Brazil.^15^ Practical Approach to Care Kit is equivalent to the Primary Care 101 guidelines that are also used in South Africa. Within the PACK guidelines, primary care workers are guided through first-line treatments for depression, substance misuse, psychosis and dementia. A training manual was developed, which provided some expansion of the PACK guidelines and also a logbook of completed case-based discussions, with space to record feedback and agreed development points. Training was delivered in weekly 1-h sessions at the clinic before the day started at 07:30. The format was a group discussion, case-based discussions based on actual patients seen in the clinic and some role plays. Additional catch-up sessions were provided when staff missed training because of leave and other commitments.

Participants were asked to bring cases they had seen in their primary care work for discussion to the training sessions. The remit for these cases was broad, including, for example, a patient in denial about an HIV diagnosis, as well as cases that fit into the common mental disorder topics such as depression.

A trainer’s guide was developed and tested during the programme, which provides questions to ask the group to stimulate discussion and guide to facilitating case-based discussions. The training was facilitated by the mental health care nurse, initially with support from the psychiatrist who attends the clinic and the first author. This support was gradually withdrawn as the confidence of the mental health care nurse improved.

In addition, primary care nurses were timetabled to the mental health nurse’s clinic once every 2–3 weeks to perform supervised patient interviews. Primary health care doctors were also invited to the training sessions, but few were able to attend and no doctors completed the course.

Although not formally evaluated here, in parallel to the training programme, workshops on mental health awareness were held for clinic support staff, including reception staff, domestic staff and nursing assistants. These sessions focused on demystifying mental illness and allowing place for staff to discuss their experiences. The aim of this intervention was reduction in stigma associated with mental illness and involvement of all clinic staff in improving the care of patients with mental illness.

### Data collection

Data were collected before the training began and again at a predetermined evaluation point, 4 months into the training programme. The training programme was intended to continue after the evaluation point as a supervision system. All participants completed a self-administered questionnaire before and after the training. They rated themselves on their competence at diagnosing and treating common mental disorders, as well as some core skills for mental health, such as mental state examination, on a Likert scale ranging from 1 to 5, where 1 = do not agree at all, 2 = do not agree, 3 = unsure, 4 = agree, and 5 = strongly agree.

This questionnaire was adapted with permission from the Programme for Improving Mental Health Care (PRIME) self-rated competence questionnaire.^16^

All referrals from the PHC team to the mental health care nurse at the clinic were collected for a period of 1 month prior to training and for 1 month at the defined evaluation point 4 months later. The number and quality of the referrals were assessed against the PACK guidelines.

Group semi-structured interviews were performed by the first author before devising the training programme as part of a learning needs assessment and to identify potential barriers to primary care nurses using the training content in their everyday work. An interview guide was used for this purpose. A qualitative feedback form was also completed after 2 months of the training for ongoing improvement of the sessions. Case-based discussions also provided a useful vehicle for qualitative feedback on how the participants were progressing in their assessment of patients. Participants could feed back difficulties that they had with performing their new role in mental health care, both in terms of their own skills and knowledge and the set-up of services.

Questionnaires and qualitative feedback from participants were collected specifically for this study. Other data on referrals and number of patients devolved were already collected at a district level as part of routine monitoring.

### Data analysis

Quantitative analysis was performed using basic descriptive statistics. Questionnaires at the evaluation point were compared with questionnaires completed before training. The number of referrals was also calculated and the percentage compliance with current best practice guidelines before and after the training programme. As the sample size was small, it was decided not to perform statistical analysis for significance on either the questionnaire or referral data.

Qualitative analysis of the semi-structured interviews and feedback forms was performed using a thematic approach.

### Ethical considerations

Ethics approval was obtained from the Higher Research Ethics Committee of the University of Stellenbosch (Ref no N16/11/144). Protocol was approved by the University of Stellenbosch Health Research Ethics Committee (Ethics Ref: N16/11/144).

## Results

**FIGURE 1:**
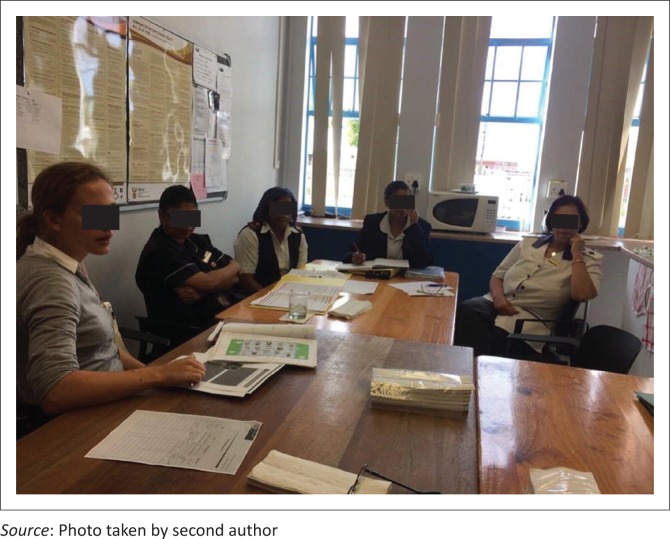
An early morning training session in action with the primary health care nurses.

**FIGURE 2:**
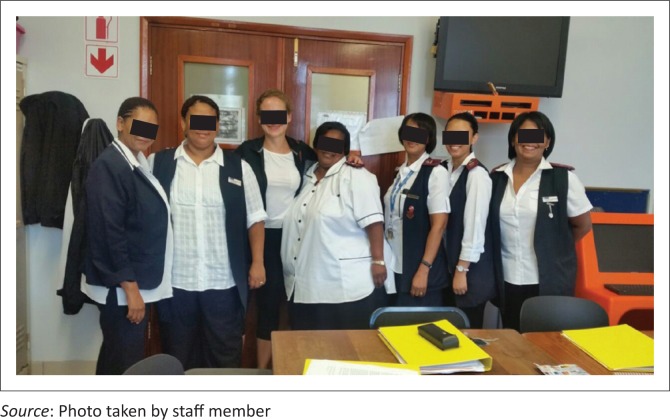
Some of the study participants with the first author.

Fourteen participants consented and were included. Of these, nine were PHC nurses. One participant was a professional nurse from the local Youth Correctional Centre. Other participants included assistant nurses and health promoters. Other staff members who were not included in the evaluation but attended some of the training included PHC nurses from surrounding clinics, student nurses and a medical intern. One PHC doctor attended four sessions before the evaluation point. All participants were female (see Figures 1 and 2).

### Questionnaire data

**FIGURE 3:**
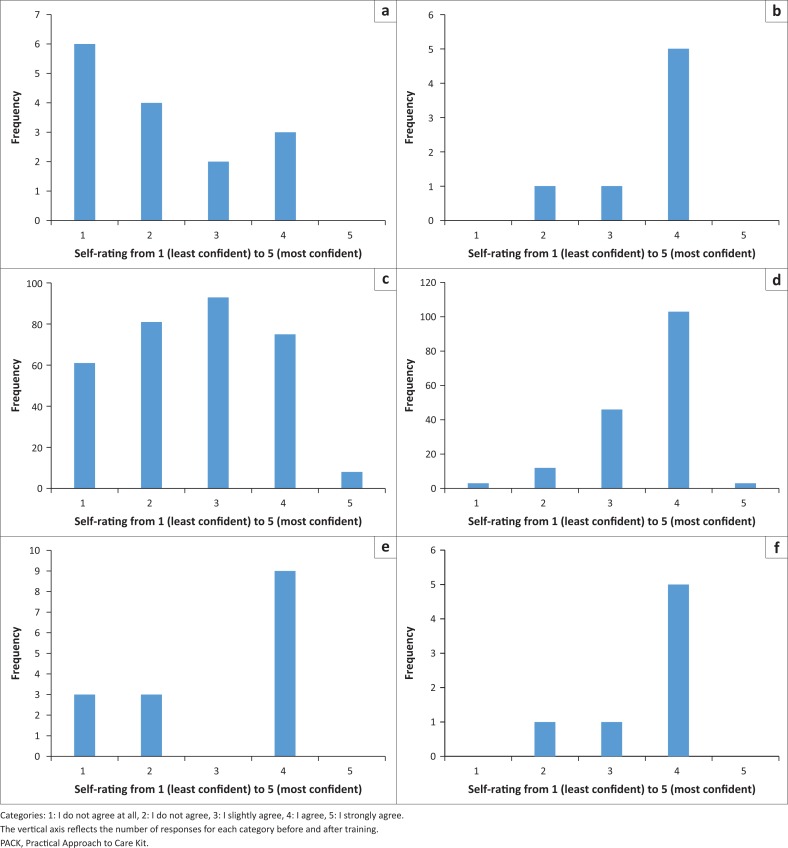
Participants’ self-rated competence across all questions. (a), I can assess (in accordance with PACK) psychosis *before training*; (b), I can assess (in accordance with PACK) psychosis *after training*; (c), Self-rating across all questions *before training*; (d), Self-rating across all questions *after training*; (e), I can assess (in accordance with PACK) depression *before training*; (f), I can assess (in accordance with PACK) depression *after training*.

A self-rated competence questionnaire was used before training and at the predefined evaluation point 4 months into training. At the evaluation point, all core topics had been covered. Supervision using case-based discussions continued after the evaluation point. Four of the participants did not complete a post-evaluation questionnaire. A sample of the questionnaire data is shown in Figure 3.

### Referrals

The number of referrals from primary care nurses to the mental health nurse decreased following the training programme. However, the total number of referrals is small and the month of December was very disrupted by staff leave and smaller patient numbers. All referrals received from training participants after training were of high quality and much more considered than before training. All three referrals included mental state examinations, which primary care nurses had not included in any referrals before (see Tables 1 and 2).

**TABLE 1:** Total referrals to the mental health nurse.

Total referrals	Before (September 2016)	After (December 2017)
Number of referrals	15	10
Source	6 from PHC nurses 7 from PHC doctors 1 from hospital 1 from physiotherapist	7 from PHC nurses 3 from PHC doctors
Percentage (%) compliance with PACK guidelines	6%	30%

PHC, primary health care; PACK, Practical Approach to Care Kit.

**TABLE 2:** Referrals to the mental health nurse only from participants who had attended the training.

Referrals	Before (September 2016)	After (December 2017)
Number of referrals	5	3
Source	2 PHC nurses at study site 3 PHC nurses at correctional services facility	2 PHC nurses at correctional services facility 1 from PHC nurses at study site
Percentage (%) compliance with PACK guidelines	0%	100%

PHC, primary health care; PACK, Practical Approach to Care Kit.

### Qualitative feedback

The four major themes that emerged before the intervention included the following:

- Mental health is currently ‘separate’.

‘We need to adjust in our minds and think back to previous training a long time ago.’ (PHC nurse, 43 years old, female)

This was concordant with current practice, which was to send patients complaining of mental health symptoms directly to the mental health nurse, without an assessment in primary care.

- Mental health patients are a big demand on nurse’s time.

‘We need to be able to spend time talking. Cannot “prescribe and go”.’ (PHC nurse, 48 years old, female)

- Mental health patients ‘cannot wait’.

‘That they are unable to wait in the queue for primary health care and it is easier for the smooth running of the clinic if patients are sent straight into the mental health sister’s waiting area.’ (PHC nurse, 51 years old, female)

- Lack of confidence and ‘feeling uncomfortable’ with mental health assessments.

‘We do not feel comfortable with mental health.’ (PHC nurses)

During training, a theme that emerged was that mental health nurses were sometimes afraid to say the ‘wrong thing’ during consultations with mental health patients. In addition, they often knew the main symptoms they were looking for, but not what questions to ask to elicit them. In response to the question, ‘When did you last have training in mental health?’, participants replied:

‘I lost most knowledge due to lack of practice and in service trainings.’ (PHC nurse, 55 years old, female )

‘I want to learn more about mental health.’ (PHC nurse, 38 years old, female )

The three major themes that emerged during the intervention included the following:

- Training was very useful, particularly in the context of clinical supervision.
  Feedback recorded on a written feedback form given out after 2 months of training was that overall the training was mostly ‘very useful’ or ‘quite useful’. Participants would like more opportunities to do supervised cases and sit in with the mental health nurse as this was the part of the training often neglected in favour of clinical commitments. Primary care nurses preferred in-service training over time compared with once-off, off-site courses.
- The discussions did foster some innovation within the team.
  One nurse from the local correctional centre designed a new pro forma for mental health assessment, based on what she had learnt, to improve her referrals to the mental health nurse.
- Engagement in the training programme increased over time.

Participants became more comfortable with discussing cases and answering questions.

## Discussion

A recent Lancet systematic review on mental health training of health workers in Africa identified as a key knowledge gap the evaluation of training that facilitates implementation of integrated mental health systems.^14^ This article describes a feasible and low-cost example of a training programme that provides a mechanism to encourage ‘task-shifting’ of first-line mental health care to primary care nurses, establishes supervision systems between primary and secondary care and develops teaching and leadership skills amongst mental health nurses. However, when considering the outcomes of this evaluation, the study is limited by small participant numbers, lack of control group and short-term outcome assessments.

The format of the training programme appears unusual in the context of other published similar interventions. On systematic review, Germaine et al. found that all identified educational interventions for mental health workers in Africa employed some didactic methods of teaching. Only two studies employed a ‘training the trainers’ approach, which was an important component of this intervention.

The implementation of this model of training in a PHC clinic was well received and proved very feasible. Some of the advantages of the programme identified included its long-term nature, its role in providing development opportunities for mental health nurses and its use as a system for supervision as well as training. This is in line with previous work that showed that bringing an educational project into the health system was perceived as evoking a new ‘identity’ for health staff, as educators and collaborators.^17^ There is a sense of an enhanced ‘status’ for the clinic as a result of being a recognised site for training, with the nurses’ own professional learning most likely being enhanced as a result of supervising others.^17^

While interventions that provide short, intensive training programmes can provide large numbers of people with high quality training, there is little evidence that it translates into a change of clinical practice. Similarly, once-off training sessions have also not resulted in a change in practice in the Eden district. One reason for this may be that mental health has some differences from other areas of chronic disease care. Historically, there has been a separation of psychiatry from the rest of medicine in South Africa.^9^ Integration will therefore require a change in attitude, perception and behaviour, as well as improving knowledge of mental health conditions, in line with international recommendations towards having an interdependent systems approach in health.^18^ Subsequently, a longer term training programme with more room for follow-up, feedback and reinforcement over time has a particular strength.

In-service training also has potential as a vehicle for putting staff supervision systems into clinics and developing the skills needed to supervise other staff in mental health care. If task-sharing has to succeed, mental health nurses and psychiatrists will need to take more of a role in teaching and supervising others. The mental health nurses in this intervention needed more support in their roles as trainers than was initially anticipated. Two ‘training the trainer’ sessions as well as ongoing one-on-one mentoring were provided. If the task-sharing model has to succeed, mental health nurses and specialists also need to be capacitated in their role as supervisors, trainers and leaders.

Delivering of training through existing clinical structures has its benefits. Discussion of ‘real’ cases with staff who work together in the facility allowed staff to use training to look at how they work. For example, the referral pathway from HIV counsellors to mental health was discussed. In this way, they could help facilitate the transition towards integrated care.

There were also some potential disadvantages of the programme identified, which included the following:

- Mental health nurses as tutors sometimes lack confidence and authority in teaching.
- The intervention is relatively resource-intensive in terms of time and staff available for multiple sessions, compared with delivering a lecture style session to more participants. However, it is less resource-intensive in the sense that it makes use of existing staff members and does not impact heavily on clinical time.
- The quality of training will vary depending on teaching skills and knowledge of the individual mental health nurse. Use of the ‘trainer’s guide’ and the PACK guidelines aim to provide a baseline level of quality. However, the ability to change perceptions of staff members and inspire them to use this knowledge in their work will be influenced more by the skill of the trainer.
- The challenge of engaging doctors in the programme: Doctors tend to have sessional appointments at clinics, or rotate, and so they are more difficult to access for in-service training. There may also be a perceived barrier between nurses and doctors that does not encourage learning, particularly when a nurse is delivering the training. Attendance from doctors was good when a consultant psychiatrist taught at their established clinical update meeting. If doctors and nurses could be learning together, it could help to improve communication and continuity of care within a clinic.

Some primary care nurses reported that they did not always feel that they had much to offer patients with mental health problems, and this may contribute to their reluctance to ask about mental health symptoms. Primary care nurses lack the ability to prescribe antidepressants or to refer for cognitive therapies. They reported not feeling that they had ‘the time to talk’. Keeping the provision of treatments for mental health problems in the field of ‘specialists’ may perpetuate the view that mental health is a separate sphere. If primary care nurses felt they had more treatment options to offer, they may be more engaged in screening for symptoms.

A weakness of the study was that it did not capture the impact on the service users. It was not possible to collect data on patient’s perceptions of a primary care-led service, or the training’s impact on detection rate of mental illness. There is work elsewhere that demonstrates good patient outcomes with integrated care.^19^ This will depend on the local context.

There is a risk with a ‘systems’ approach, namely that the impact of change in services on patients is lost. Task-sharing should be driven by improving detection of mental illness in patients who attend for other chronic conditions and improving access and integration of care. There are other advantages to a ‘mental health literate’ primary care team, such as a reduction in stigma. A PHC team that understands mental health problems, knows how to look for them and is comfortable asking questions about a patient’s psychosocial well-being is likely to be beneficial overall. If primary care nurses feel increasingly responsible for mental health patients but do not feel empowered to provide them with treatment options, then there is a risk they will avoid asking about mental health symptoms.

## Conclusion

The aim of the study was to develop and evaluate a locally delivered, long-term, in-service training programme to facilitate the delivery of mental health care at a primary care level. We demonstrated that such an intervention is acceptable and feasible to clinic nurses in PHC, supporting the transition into a task-sharing model of integrated mental health care. Staff engagement in discussions improved noticeably after 3 months, showing the importance of persistence with this type of intervention. The value of the process in developing the mental health nurse as a trainer and supervisor was underappreciated at the start of the programme. We expect this model to have greater sustainability in the long term than in shorter, externally driven courses. The next step will be to re-evaluate staff knowledge and practice after a longer time period.

## References

[1] World Health Organization Comprehensive mental health action plan 2013–2020 [homepage on the Internet]. 2013 [cited 2017 Sept 28]. Available from: http://apps.who.int/gb/ebwha/pdf_files/WHA66/A66_10Rev1-en.pdf

[2] World Health Assembly The global burden of mental disorders and the need for a comprehensive, coordinated response from health and social sectors at the country level [homepage on the Internet]. 2012; p. 2–5 [cited 2018 Feb 16]. Available from: http://apps.who.int/gb/ebwha/pdf_files/WHA65/A65_R4-en.pdf

[3] Kakuma R, Minas H, Van Ginneken N, et al Human resources for mental health care: Current situation and strategies for action. Lancet. 2001;378(9803):1654–1663. https://doi.org/10.1016/S0140-6736(11)61093-3 10.1016/S0140-6736(11)61093-3 22008420

[4] World Health Organization Task shifting: Rational redistribution of tasks among health workforce teams – Global recommendations and guidelines [homepage on the Internet]. WHO; 2008; p. 1–82 [cited 2018 Feb 16]. Available from: http://www.who.int/healthsystems/TTR-TaskShifting.pdf

[5] Petersen I, Lund C, Bhana A, Fisher AJ A task shifting approach to primary mental health care for adults in South Africa: Human resource requirements and costs for rural settings. Health Policy Plan. 2012;27(1):42–51. https://doi.org/10.1093/heapol/czr012 21325270 10.1093/heapol/czr012

[6] South African National Department of Health National mental health policy framework and strategic plan 2013–2020. Pretoria: National Department of Health; 2013; p. 1–60.

[7] De Kock JH, Pillay BJ A situation analysis of psychiatrists in South Africa’s rural primary healthcare settings. Afr J Prim Health Care Fam Med. 2017;9(1):a1335 https://doi.org/10.4102/phcfm.v9i1.1335 10.4102/phcfm.v9i1.1335 PMC5458564 28582989

[8] Williams D, Herman A, Stein D, et al Twelve-month mental disorders in South Africa: Prevalence, service use and demographic correlates in the population-based South African Stress and Health Study. Psychol Med. 2008;38(2):211–220. https://doi.org/10.1017/S0033291707001420 17903333 10.1017/S0033291707001420 PMC2718686

[9] Mash B, Fairall L, Adejayan O, et al A morbidity survey of South African primary care. PLoS One. 2012;7(3):e32358 https://doi.org/10.1371/journal.pone.0032358 22442666 10.1371/journal.pone.0032358 PMC3306367

[10] Moussavi S, Chatterji S, Verdes E, Tandon A, Patel V, Ustun B Depression, chronic diseases and decrements in health: Results from the World Health Surveys. Lancet. 2007;370:851–858. https://doi.org/10.1016/S0140-6736(07)61415-9 17826170 10.1016/S0140-6736(07)61415-9

[11] Horberg MA, Silverberg MJ, Hurley LB, et al Effects of depression and selective serotonin reuptake inhibitor use on adherence to highly active antiretroviral therapy and on clinical outcomes in HIV-infected patients. J Acquir Immune Defic Syndr. 2008;47(3):384–390. https://doi.org/10.1097/QAI.0b013e318160d53e 18091609 10.1097/QAI.0b013e318160d53e

[12] World Health Organization Integrating mental health into primary care: A global perspective [homepage on the Internet]. World Health Organization; 2008 [cited 2018 Feb 16]. Available from: http://www.who.int/mental_health/resources/mentalhealth_PHC_2008.pdf

[13] Cornick R, Fairall L, Carkeek E, Picken S, Wattrus C Practical Approach to Care Kit, Knowledge Translation Unit, University of Cape Town. Cape Town: Department of Health, Western Cape Government; 2017.

[14] Liu G, Jack H, Piette A, et al Mental health training for health workers in Africa: A systematic review. Lancet Psychiatry. 2016;3(1):65–76. https://doi.org/10.1016/S2215-0366(15)00379-X 26772066 10.1016/S2215-0366(15)00379-X

[15] Knowledge Translation Unit, University of Cape Town Preliminary results [homepage on the Internet]. [cited 2017 Jan 04]. Available from: http://knowledgetranslation.co.za/research/current-research-trials/primary-care-101-trial/

[16] Adapted with permission from the PRIME self-report competence evaluation [homepage on the Internet]. [cited 2017 Jan 04]. Available from: http://www.prime.uct.ac.za/research/tools

[17] Blitz J, Bezuidenhout J, Conradie H, De Villiers M, Van Schalkwyk S ‘I felt colonised’: Emerging clinical teachers on a new rural teaching platform. Rural Remote Health. 2014;14:2511. 24803205

[18] Frenk J, Chen L, Bhutta ZA, et al Health professionals for a new century: Transforming education to strengthen health systems in an interdependent world. Lancet. 2010;376(9756):1923–1958. https://doi.org/10.1016/S0140-6736(10)61854-5 21112623 10.1016/S0140-6736(10)61854-5

[19] Araya R, Rojas G, Fritsch R, et al Treating depression in primary care in low-income women in Santiago, Chile: A randomised controlled trial. Lancet. 2003;361(9362):995–1000. https://doi.org/10.1016/S0140-6736(03)12825-5 12660056 10.1016/S0140-6736(03)12825-5

